# Virulence arsenal of *Acinetobacter baumannii*: mechanisms driving persistence and resistance

**DOI:** 10.1007/s00203-025-04668-7

**Published:** 2026-02-02

**Authors:** K. Ayswarya, Rafwana Ibrahim, Vimal V. Veetilvalappil, Nishanth B. Bhat, Jesil Mathew Aranjani

**Affiliations:** 1 Department of Pharmaceutical Biotechnology, Manipal College of Pharmaceutical Sciences, Manipal Academy of Higher Education, Manipal, Udupi, Karnataka 576104 India; 2 Department of Pharmaceutical Chemistry, Devaki Amma Memorial College of Pharmacy, Malappuram, Chelembra, 673636 India; 3 Division of Microbiology, Department of Basic Medical Sciences, Manipal Academy of Higher Education, Manipal, Udupi, Karnataka 576104 India

**Keywords:** Acinetobacter baumannii, Virulence factors, Biofilm, Outer membrane protein A, Lipid A modification, Antimicrobial resistance

## Abstract

*Acinetobacter baumannii* is a Gram-negative opportunistic pathogen and a major cause of healthcare-associated infections, including ventilator-associated pneumonia, bacteraemia, meningitis, and urinary tract infections. Its persistence in hospital environments is due to its ability to survive desiccation, resist disinfectants, and colonize both biotic and abiotic surfaces. Virulence in *A. baumannii* is largely associated with structures such as Csu pili, biofilm-associated protein (Bap), and outer membrane protein A (OmpA), which enable surface attachment, biofilm formation, and host cell damage. The production of extracellular polysaccharides and quorum sensing further enhance biofilm development. Iron uptake systems support bacterial growth even under iron-limited conditions within the host. Resistance to polymyxins often results from lipid A modifications regulated by the PmrCAB operon and *LpxL*-related genes, which also reduce immune recognition via the TLR4 pathway. Phase variation allows phenotypic changes that aid immune evasion. The highly adaptable genome of *A. baumannii* enables rapid acquisition of multiple resistance determinants, including OXA-type carbapenemases, efflux pumps, and aminoglycoside-modifying enzymes. Due to its extensive multidrug resistance, the World Health Organization lists *A. baumannii* as a critical-priority pathogen. This review aims to comprehensively examine the molecular mechanisms driving its virulence and resistance, highlighting potential therapeutic targets and strategies to combat this formidable pathogen.

## Introduction

*Acinetobacter baumannii* is a Gram-negative bacillus responsible for hospital-acquired infections across the world (Gedefie et al. 2021). The major infections caused by this pathogen include meningitis, ventilator-associated pneumonia (VAP), urinary tract infections, and bacteraemia in immunocompromised patients, leading to increased mortality and morbidity (Jiang et al. 2020). Its ability to survive on dry surfaces for extended periods facilitates persistence and transmission in hospital settings, underscoring the importance of stringent infection control measures. These include proper hand hygiene, routine disinfection of hospital environments, and isolation of infected patients to prevent outbreaks (Sherif et al. 2021; Zhang et al. 2025). Furthermore, its capacity to form thick biofilms on medical devices enhances survival and antimicrobial resistance, making it a particularly challenging pathogen to eradicate.

In recent decades, *A. baumannii* has evolved from an opportunistic pathogen of limited clinical relevance to one of the most formidable causes of hospital-acquired infections worldwide. Its remarkable ability to acquire resistance determinants and persist in healthcare environments has resulted in widespread multidrug-resistant (MDR) and extensively drug-resistant (XDR) clones. According to the World Health Organization (WHO 2024), carbapenem-resistant *A. baumannii* (CRAB) is designated as a critical-priority pathogen due to the scarcity of effective antibiotics and its association with high morbidity and mortality. Recent surveillance data indicate that carbapenem resistance in *A. baumannii* exceeds 60–70% in several hospitals in South and Southeast Asia and approaches 40% in European intensive care units (Lee et al. 2023; WHO 2024). Outbreaks are frequently documented in critical care settings, where invasive devices such as ventilators and catheters facilitate colonization and transmission. This alarming epidemiological trend highlights the urgent global need for enhanced infection control, genomic surveillance, and the exploration of alternative therapeutic strategies to mitigate the escalating threat posed by *A. baumannii*.

The COVID-19 pandemic has unfortunately contributed to a surge in antimicrobial resistance due to the widespread misuse and overuse of broad-spectrum antibiotics, particularly in settings like Lebanon. Despite a low prevalence of confirmed bacterial coinfections, a significant proportion of COVID-19 patients received antibiotics empirically, often without appropriate clinical indication. This practice has exerted antibiotic pressure leading to increased selection of resistant pathogens and highlights the urgent need for strengthened antimicrobial stewardship during and after the pandemic to mitigate long-term consequences on antimicrobial resistance (Chaaban et al. 2024; Wong et al. 2016).

The pathogen’s multidrug resistance arises from its multifaceted arsenal of defense mechanisms, including the production of β-lactamases that hydrolyze β-lactam antibiotics, alterations in penicillin-binding proteins, efflux pump activation to expel antibiotics, and modification of target sites to reduce antibiotic binding (Arroyo et al. 2011; Elkheloui et al. 2020). Its highly flexible genome, capable of acquiring resistance determinants through horizontal gene transfer, further enhances its adaptability (Sheldon and Skaar 2020; Mohamed et al. 2023). As a result, treatment options are increasingly limited (Zhu et al. 2020).

Comparative genomic studies in pathogens like *Streptococcus agalactiae* serve as powerful tools for unraveling the genetic basis of virulence, antimicrobial resistance, and pathogen evolution. For example, extensive genomic analyses of *S. agalactiae* isolates have revealed a core genome with significant plasticity, alongside an open pan-genome harboring a diverse array of virulence genes such as those encoding capsular polysaccharides (cps), adhesins (*lmb*,* fbs*), proteases (*scpB*), and toxins (*cylE*) (de Aguiar et al. 2016). These genes contribute to critical pathogenic processes including immune evasion, host cell adhesion and invasion, biofilm formation, and resistance to host defense peptides.​Such studies also identify antimicrobial resistance determinants, including genes conferring resistance to macrolides, tetracyclines, and other antibiotics, highlighting emerging resistance patterns within clonal populations. Insights into the distribution and diversity of virulence and resistance genes across isolates illustrate how genomic plasticity facilitates the adaptation and persistence of pathogens in different host and environmental niches.​By comparing genomes of pathogenic and non-pathogenic strains, researchers can pinpoint genetic factors that distinguish virulent lineages, informing the development of targeted interventions. This comparative framework not only enhances understanding of pathogen biology but also guides the design of antivirulence strategies and novel therapeutics that disrupt key pathogenic mechanisms without exerting antibiotic selection pressure.

Conventional antibiotics often fail to eradicate biofilm-associated cells, which are shielded from both antimicrobial agents and the host immune response (Cook-Libin et al. 2022; Upmanyu et al. 2022). This biofilm-mediated resistance contributes to prolonged hospitalizations, increased mortality, and greater healthcare costs (Moffatt et al. 2010). In many clinical cases, colistin serves as the last-resort therapy; however, emerging reports of colistin-resistant strains, often linked to modifications in the lipid A component of lipopolysaccharides, further complicate treatment (Roy et al. 2022; Wang et al. 2025).

This review examines the diverse virulence factors of *A. baumannii*, focusing primarily on their contributions to pathogenesis, immune evasion, biofilm formation, and antibiotic resistance. It also highlights how these factors collectively drive its persistence, adaptability, and clinical severity as a multidrug-resistant pathogen.

## Virulence factors

**Fig. 1:**
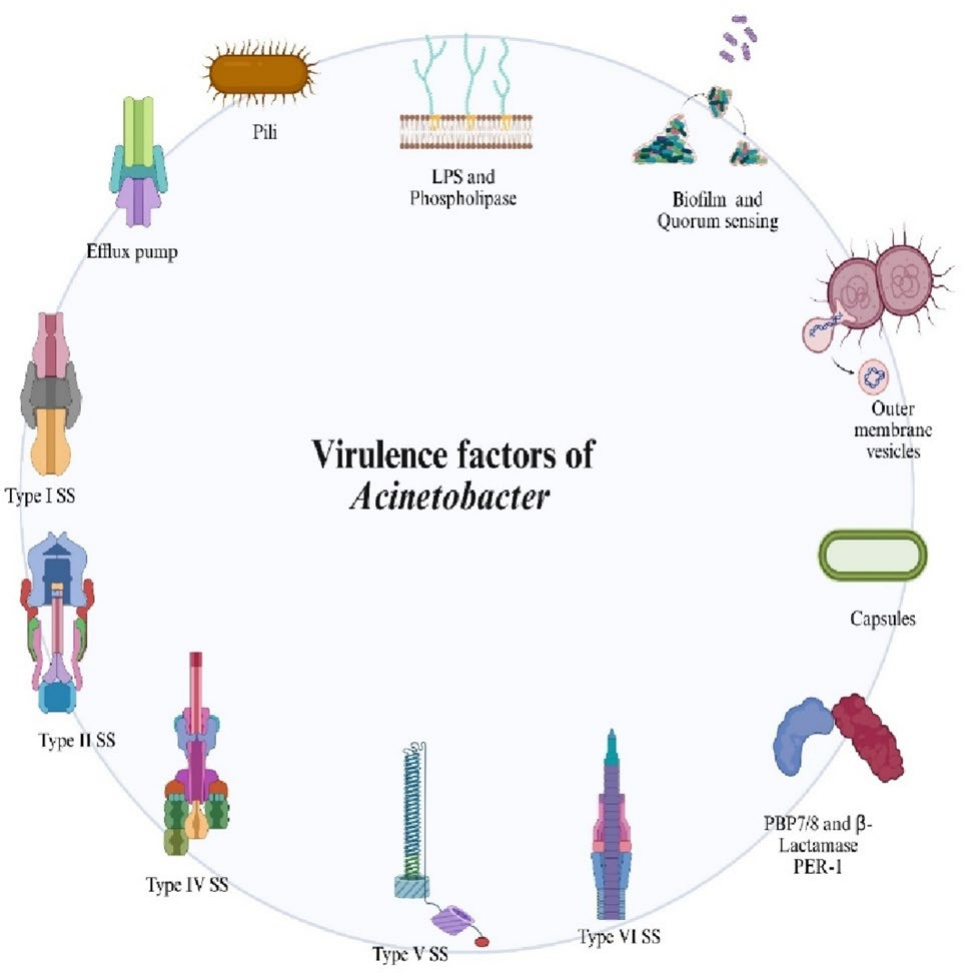
Virulence factors of *Acinetobacter* spp. A schematic representation of the major virulence determinants contributing to the pathogenicity of *Acinetobacter* species. These include pili, lipopolysaccharides (LPS), phospholipases, outer membrane vesicles, capsules, biofilm formation and quorum sensing, peptidases and proteases (e.g., OmpA, Pgp1), secretion systems (Type I, II, IV, V, and VI), and efflux pumps. Together, these factors facilitate host colonization, immune evasion, persistence, and multidrug resistance. Created in BioRender. Aranjani (2025) https://BioRender.com/9fwuslj

*A. baumannii* has developed multiple virulence traits that function collectively as a survival toolkit. This allows the pathogen to flourish in the difficult settings of contemporary healthcare. *A. baumannii* can establish invasive infections. Simultaneously evade the immune system, survive antibiotic treatment, and persist for long periods of time on inanimate and animate surfaces (Fig. 1). The capacity of *A. baumannii* to emerge as a formidable clinical threat is due to the flexibility of its virulence traits, particularly in cases of immunocompromised patients and those exposed to multiple invasive procedures.

### Adherence

#### Outer membrane proteins (OMPs)

Among the most extensively characterized virulence determinants of *A. baumannii* are its outer membrane proteins, particularly outer membrane protein A (OmpA) and biofilm-associated protein (Bap). OmpA, encoded by the *ompA* gene, plays a multifaceted role in pathogenicity. It mediates the initial adhesion of the bacterium to host epithelial cells, establishing the foundation for colonization and infection. Beyond its adhesive function, OmpA promotes cellular aggregation and strengthens the structural integrity of biofilms formed on both biotic and abiotic surfaces (Gaddy and Actis 2009; Smani et al. 2013). Functionally, OmpA also contributes to immune evasion by inducing apoptosis in host cells, interfering with complement activation, and protecting bacterial communities within biofilms from phagocytic clearance. These activities collectively enhance bacterial persistence under immune and antibiotic pressure (Yehya et al. 2025).

In contrast, Bap, encoded by the *bap* gene, primarily acts as a high-molecular-weight scaffolding component that facilitates intercellular adhesion and stabilizes the biofilm extracellular matrix (Loehfelm et al. 2008; Nie et al. 2020; Tiku et al. 2021; Kakavan et al. 2025). Disruption of *bap* expression has been shown to significantly diminish biofilm biomass and reduce the capacity of *A. baumannii* to persist on medical devices and host surfaces.

While both proteins contribute to biofilm formation, their mechanisms are distinct yet complementary: OmpA enhances surface attachment and immune modulation, whereas *Bap* fortifies the biofilm architecture and ensures long-term stability. Together, these virulence factors underpin *A. baumannii*’s ability to resist disinfection, evade immune defenses, and withstand antibiotic therapy, thereby contributing to its clinical recalcitrance and persistence in healthcare environments (Brossard and Campagnari 2012).

#### Biofilm formation and regulation

The ability to assemble extensive biofilms is central to *A. baumannii*’s persistence in clinical environments. Biofilms are complex bacterial communities embedded in a self-produced matrix of extracellular polymeric substances (EPS), including polysaccharides, proteins, and extracellular DNA. Within this protective matrix, *A. baumannii* exhibits high resistance to antibiotics and reduced susceptibility to immune clearance (Gaddy and Actis 2009; Smani et al. 2013).

Biofilm formation is a dynamic and tightly regulated process influenced by environmental cues. Two-component regulatory systems (TCSs) such as *BfmRS* and *PmrAB* play pivotal roles in coordinating surface attachment, biofilm maturation, and resistance mechanisms. *BfmRS* primarily controls the expression of adhesion-related genes essential for biofilm initiation and development. The *PmrAB* system, best known for polymyxin resistance through lipid A modification, also contributes to exopolysaccharide production and modulation of biofilm architecture under specific environmental conditions (Olaitan et al. 2014; Tipton et al. 2015; Eze et al. 2018; Wijers et al. 2021). Together, these regulatory networks fine-tune virulence gene expression to optimize survival in both host and environmental niches.

In healthcare settings, biofilm formation extends beyond biotic surfaces. *A. baumannii* readily develops biofilms on abiotic materials such as the plastic surfaces of ventilators, catheters, and other indwelling medical devices. These biofilms act as reservoirs for persistent bacteria, promoting chronic and recurrent infections while resisting standard disinfection procedures (Arroyo et al. 2011; Beceiro et al. 2014). Biofilm communities display altered metabolism, differential gene expression, and an enhanced ability to acquire additional resistance determinants, further complicating eradication efforts.

Clinically, biofilm-associated infections often necessitate the removal or replacement of contaminated devices, prolonged antibiotic therapy, and strict infection control measures. Such infections are linked to increased hospital stays, higher healthcare costs, and greater patient morbidity and mortality. Outbreaks frequently arise from the organism’s ability to survive and disseminate through biofilm formation on medical equipment and environmental surfaces. This resilience underscores the urgent need to develop therapeutic strategies that disrupt biofilm structure and prevent its role in chronic infection and transmission (Nguyen et al. 2021).

#### Pili and fimbriae

Surface appendages such as Csu pili, which are assembled from proteins encoded by the *csuA/B* gene cluster, are fundamental to the initial adhesion stage of biofilm formation. The Csu pili are hair-like filaments that mediate attachment to surfaces. The integrity and assembly of these pili rely on several additional genes, including *csuC*. These are key structural and regulatory components for pilus function and promote effectiveness in both attachment and biofilm maturation (Tomaras et al. 2003; Gedefie et al. 2021).

**Fig. 2:**
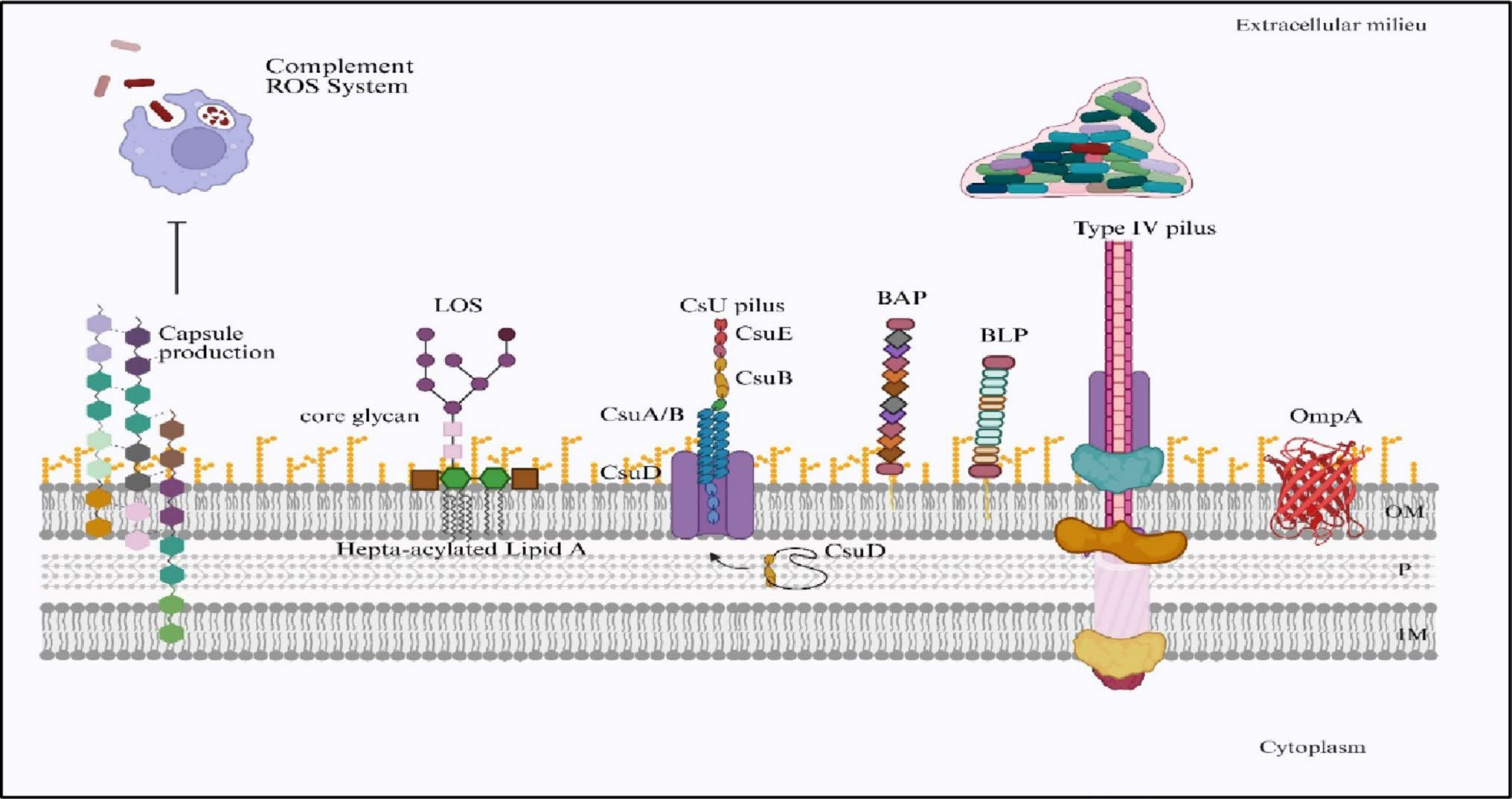
Cell-surface virulence factors in *Acinetobacter spp*. Schematic illustration of key surface components that contribute to virulence and persistence in *Acinetobacter* species. The capsule provides protection against complement attack and reactive oxygen species. Lipooligosaccharides (LOS) with core glycan and hepta-acylated lipid A modifications enhance resistance to host immune defenses. The Csu pilus system (CsuA/B, CsuB, CsuE, CsuD) supports adhesion and biofilm initiation on abiotic surfaces. Biofilm-associated proteins (BAP) and biofilm-associated lipoproteins (BLP) facilitate biofilm stability and maturation. Type IV pili mediate motility, surface attachment, and DNA uptake, while outer membrane protein A (OmpA) plays a central role in adhesion and invasion. These structures collectively strengthen environmental survival, colonization, and pathogenic potential. Created in BioRender. Aranjani (2025) https://BioRender.com/9fwuslj

These pili not only initiate colonization but also contribute to long-term persistence in healthcare settings. Surfaces that are colonized by Csu pili-mediated biofilms have emerged as hotspots for persistent reservoirs of *A. baumannii*. This complicates eradication efforts and increases the likelihood of transmission between patients (Lee et al. 2008). In the intensive care unit there is a significant correlation between the presence of these pili structures and recurrent outbreaks of *A. baumannii* that is demonstrated by the Clinical studies (Vijayakumar et al. 2022) (Fig. 2).

### Resistance to host immune responses

*A. baumannii* has developed several ways to survive and grow even when attacked by the host’s immune system. These include structural features like the capsule and lipopolysaccharide (LPS) that protect the cell. As well as systems such as efflux pumps that help the bacterium to resist harmful substances. Together, these mechanisms make *A. baumannii* a challenging pathogen that is difficult to treat in hospitals. The following section explains each of these factors in detail to show how they help the bacterium escape the body’s immune defences.

#### Capsular polysaccharides and immune evasion

The polysaccharide capsule forms a thick, slippery layer that envelopes the surface of *A. baumannii*. This structure plays an important role in protecting the bacterium from being identified, engulfed, and destroyed by the host’s immune cells, such as macrophages and neutrophils. By masking conserved surface antigens and interfering with the process of opsonization, the capsule prevents immune cells from recognizing the bacterium, thereby helping it evade phagocytosis (Geisinger and Isberg 2017; Monem et al. 2020). In addition to this protective function, the capsule also interferes with the host’s complement system. This is a chain of serum proteins responsible for clearing bacteria from the body. Normally, complement activation results in opsonization and the formation of the membrane attack complex, leading to bacterial lysis and removal. However, the capsule of *A. baumannii* prevents proper binding and activation of these complement components. This allows the bacterium to evade complement-mediated destruction (Monem et al. 2020; Bharadwaj et al. 2022). The genes responsible for capsule formation in *A. baumannii* are organized within the *kps* gene cluster, which regulates the synthesis and export of polysaccharide chains that form this protective layer. Importantly, variability in capsule composition among strains further complicates immune recognition (Singh et al. 2019; Taylor et al. 2004), allowing persistence and recurrent infections even in patients previously exposed to the bacterium (Fig. 2).

The role of the capsule in clinical outcomes is profound. Capsule-deficient mutants show dramatically reduced survival in animal models, and heavily encapsulated strains are linked to worse patient prognoses and persistent infections. The capsule also plays a role in biofilm formation. They provide a scaffold for other virulence factors and enhance surface attachment. Biofilm-associated capsule material can further impair the penetration of immune molecules and antibiotics, making infections both chronic and resistant to clearance (Monem et al. 2020).

#### Lipopolysaccharide (LPS) modifications

LPS, which is found in the outer leaflet of the Gram-negative cell wall, acts as both a structural barrier and a potent immunomodulator. The lipid A moiety within LPS is a classical activator of the host’s innate immune system. It binds to Toll-like receptor 4 (TLR4) on immune cells, alerting the host to bacterial invasion and driving the production of pro-inflammatory cytokines (Matsuura et al. 2013, Alexander and Rietschel 2001).

*A. baumannii* has developed advanced mechanisms to escape detection by the host immune system. One of the most effective among them is the targeted modification of its lipopolysaccharide (LPS). A key aspect of this defense involves changes to lipid A, the biologically active part of LPS. These changes are brought about by the action of genes such as *lpxA*, *lpxC*, and *lpxD*, along with modifying enzymes like *PmrC*. The bacterium alters lipid A by adding chemical groups such as phosphoethanolamine or by modifying its acylation pattern. These structural alterations help *A. baumannii* reduce immune recognition and improve its ability to survive under hostile conditions within the host. (Zhang et al. 2007; Henry et al. 2012; Pelletier et al. 2013; Carretero-Ledesma et al. 2018; Bharadwaj et al. 2022; Kim et al. 2022b; Romano et al. 2024). Such structural remodeling fundamentally alters the physicochemical properties of the bacterial surface, shaping its interactions with the host immune system.

A primary consequence of these lipid A modifications is reduced immune recognition. Normally, lipid A is sensed by the Toll-like receptor 4 (TLR4)–MD2 complex, triggering robust proinflammatory cytokine release and recruitment of neutrophils and macrophages. However, changes in acylation patterns or the addition of phosphoethanolamine disturb the exact molecular interaction needed for proper activation of the TLR4–MD2 complex (Maeshima and Fernandez 2013; Rocha et al. 2016). Due to this weakened interaction, the MyD88 and TRIF signaling pathways are less effectively activated. As a result, the production of cytokines decreases, leading to a weaker inflammatory response (Kang and Lee, 2011). This suppressed immune activity allows *A. baumannii* to survive and establish infection within the host.

In parallel, LPS modifications enhance resistance to cationic antimicrobial peptides (AMPs), key effectors of the innate immune system produced by epithelial cells and neutrophils. The addition of phosphoethanolamine increases the net positive charge of lipid A, thereby reducing the electrostatic attraction between the negatively charged bacterial surface and positively charged antimicrobial peptides (AMPs) (Sun and Shang 2015, Scott and Hancock 2000). This charge repulsion prevents AMPs from inserting into and disrupting the bacterial membrane, effectively neutralizing one of the host’s most potent early defense mechanisms.

These structural alterations also increase bacterial survival in the bloodstream during bacteremia by enhancing resistance to innate immune effectors (Needham and Trent 2013). Modified lipid A and LPS hinder efficient deposition of complement components and assembly of the membrane attack complex, thereby reducing complement-mediated lysis (Matsuura 2013). As a result, strains carrying highly modified LPS variants are better able to persist in blood, significantly increasing the likelihood of sustained bacteremia and progression to life‑threatening septicemia (Gabarin et al. 2021).

Genetic studies have demonstrated that *A. baumannii* clinical isolates harboring mutations in genes responsible for lipopolysaccharide (LPS) biosynthesis and modification exhibit profoundly altered virulence phenotypes. Key genes implicated include lpxA, lpxC, and lpxD, which are involved in lipid A biosynthesis, as well as *lpsB* and *lptD*, which participate in core oligosaccharide assembly and LPS transport, respectively. Mutations or inactivation of the lpx genes lead to the complete loss of lipid A, rendering strains deficient in LPS production, often referred to as “LPS-null” mutants. Such alterations confer resistance to polymyxins, particularly colistin, by eliminating the antibiotic’s target site (Moffatt et al. 2010; Henry et al. 2012). Similarly, modifications mediated by genes such as pmrA, pmrB, and eptA can lead to the addition of phosphoethanolamine to lipid A, decreasing its negative charge and thus reducing binding affinity to cationic antimicrobial peptides (Pelletier et al. 2013; Zhang et al. 2007). While these genetic adaptations enhance survival under selective pressures such as strong immune responses and antibiotic exposure, they often impose fitness costs, including reduced membrane integrity and attenuated virulence in the absence of such pressures (Romano et al. 2024). These findings underscore the evolutionary trade-offs shaping the adaptive landscape of *A. baumannii* in clinical environments.

While such lipid A remodeling fortifies the bacterial surface against host detection and killing, *A. baumannii* does not rely solely on passive structural defenses. It also deploys active transport systems capable of ejecting a wide range of harmful compounds from the cell. These efflux pumps function as molecular bouncers, working in parallel with LPS modifications to neutralize threats posed by both antibiotics and host immune molecules.

#### Efflux pumps: molecular shields in host–pathogen interaction

Along with modifying its surface and releasing virulence factors, *A. baumannii* also depends on advanced membrane transport systems to remove harmful substances from its cells. These systems actively pump out not only antibiotics but also toxic molecules produced by the human immune system, such as cationic antimicrobial peptides (e.g., LL-37 and human β-defensins), bile salts, and long-chain fatty acids. This dual functionality allows the bacterium to survive oxidative and immune-mediated stress during infection (Ayoub et al. 2020; Lin et al. 2014; Rosenfeld and Shai 2012).

Among these, the most well-characterized are the AdeABC and AdeIJK efflux pump systems, which are central to the pathogen’s multidrug resistance and immune evasion strategies (Xie et al. 2025).

The AdeABC efflux pump, encoded by *adeA*,* adeB*, and *adeC*, has a remarkably broad substrate profile, enabling the extrusion of diverse antimicrobial agents. Crucially, this system also contributes to resistance against host-derived cationic antimicrobial peptides (AMPs), functioning as an active barrier to direct killing by immune cells. The overexpression of AdeABC is frequently detected in multidrug-resistant clinical isolates and is strongly associated with increased bacterial survival during infection as well as persistence in hospital environments (Coyne et al. 2010).

Similarly, the AdeIJK system, encoded by *adeI*,* adeJ*, and *adeK*, plays an important role in reducing the intracellular accumulation of antibiotics and toxic metabolites. By broadening resistance to multiple antimicrobial stressors, this efflux pump allows *A. baumannii* to withstand diverse challenges posed by both therapeutic interventions and host immunity (Damier-Piolle et al. 2008; Coyne et al. 2010; Hassan et al. 2021).

These efflux systems do not work at a constant level but adjust their activity based on environmental signals such as exposure to antibiotics or host immune molecules. This flexible regulation allows *A. baumannii* to fine-tune its resistance during infection, adapting to changes in antimicrobial pressure or the host’s defense strategies (Fig. 2).

Importantly, the role of efflux pumps in immune evasion goes beyond simply removing harmful compounds. Studies indicate that they can also alter the structure and charge of the bacterial cell envelope, making the bacterium less vulnerable to damage by antimicrobial peptides and reducing the chances of being marked for destruction through opsonization. These combined effects show that efflux pumps contribute not only to antibiotic resistance but also play a key part in strengthening the overall virulence of *A. baumannii* (Damier-Piolle et al. 2008; Coyne et al. 2011).

#### Clinical relevance and integration of immune evasion mechanisms

Taken together, these immune evasion strategies, including capsular shielding, LPS modification, and multidrug efflux, provide *A. baumannii* with a formidable set of tools for survival within the human host. This synergy is reflected in the clinical outcomes of patients infected with highly resistant strains: infections are often protracted, hard to treat, and associated with high mortality (Akoolo et al. 2022, Shi et al. 2024). These same mechanisms compromise the efficacy of many current antibiotic regimens as well as the effectiveness of host-derived immune defenses, highlighting why *A. baumannii* is considered one of the most challenging hospital pathogens worldwide (Shi et al. 2024).

Understanding these mechanisms is essential for developing new treatment strategies. Approaches such as targeting the bacterial capsule, inhibiting LPS-modifying enzymes, and blocking efflux pumps are being explored as supportive therapies alongside existing antibiotics. These strategies aim to restore the body’s natural immune defenses and improve patient recovery, especially in the face of rising antibiotic resistance (Harding et al. 2018; Wang et al. 2025).

### Secretion systems

**Fig. 3:**
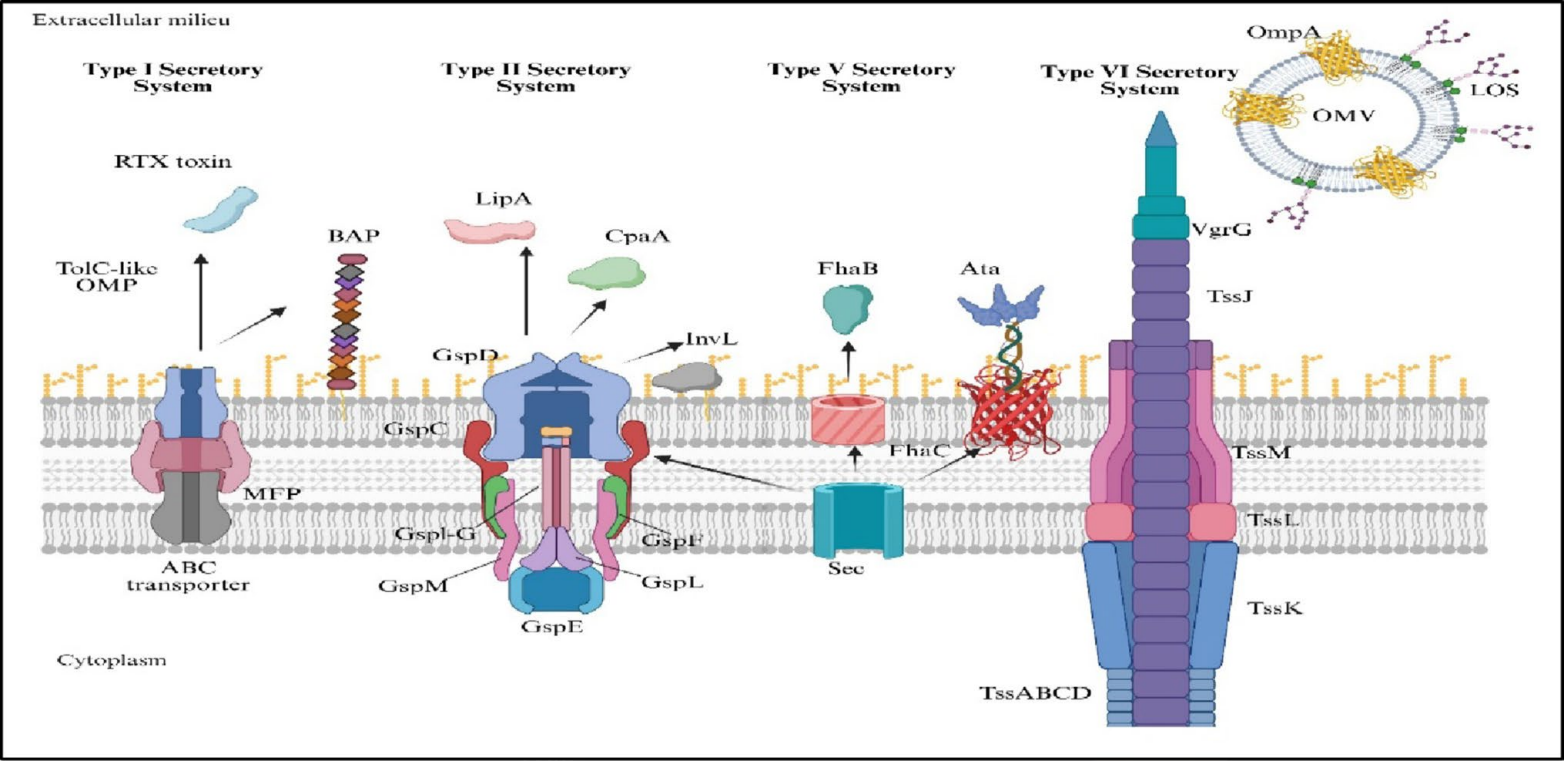
Schematic representation of major protein secretion systems in Acinetobacter species and their associated virulence determinants. The diagram illustrates Type I (T1SS), Type II (T2SS), Type V (T5SS), and Type VI (T6SS) secretion systems, as well as outer membrane vesicles (OMVs), highlighting their structural components and secreted effector molecules involved in pathogenicity. The T1SS transports proteins such as the RTX toxin and biofilm-associated protein (Bap) through a TolC-like outer membrane protein in conjunction with an ABC transporter–MFP complex. The T2SS machinery (GspD–GspM) mediates the secretion of enzymes and virulence factors including LipA, CpaA, and InvL. The T5SS (autotransporter pathway) exports adhesins such as FhaB and Ata via Sec translocation and β-barrel assembly proteins such as FhaC. The T6SS apparatus (TssABCD, TssK, TssL, TssM, TssJ, VgrG) functions as a contractile nanomachine enabling effector delivery to competing bacteria or host cells, contributing to interbacterial competition and virulence. Outer membrane vesicles enriched in OmpA and LOS further facilitate immune modulation, adhesion, and horizontal transmission of virulence factors. Collectively, these secretion systems play pivotal roles in host colonization, biofilm development, immune evasion, cytotoxicity, and environmental persistence in Acinetobacter spp. Created in BioRender. Aranjani (2025) https://BioRender.com/9fwuslj

*A. baumannii* relies on specialized secretion systems to interact with both its microbial competitors and host organisms, playing leading roles in pathogenesis, persistence, and adaptability. The main types of these systems are the type VI secretion system (T6SS) and type IV secretion system (T4SS), each of which has distinct and multifaceted functions in terms of survival and virulence (Yehya et al. 2025) (Fig. 3).

#### Type VI secretion system (T6SS)

The type VI secretion system (T6SS) is a highly evolved, contractile nanomachine that operates as a molecular syringe, delivering effector proteins into target cells in a manner analogous to bacteriophage tail contraction. Structurally, it comprises a sheath and inner tube complex anchored in the bacterial envelope that is capable of propelling effector-loaded tubes directly into adjacent cells. In *A. baumannii*, this versatile system mediates two critical biological processes: interbacterial competition and manipulation of the host environment.

In competitive environments such as the human microbiota or hospital surfaces, *A. baumannii* is constantly challenged by other bacterial species. The Type VI Secretion System (T6SS) gives it a strong survival advantage by injecting toxic effector proteins into nearby Gram-negative bacteria. These proteins can damage the target cell’s wall, disrupt its membrane, or interfere with essential metabolic functions, allowing *A. baumannii* to dominate its surroundings (Carruthers et al. 2013). This targeted killing enhances *A. baumannii*’s ability to dominate mixed microbial communities, particularly in polymicrobial infections of the lungs, wounds, or medical devices, where space and nutrients are scarce.

In addition to microbial antagonism, the T6SS also targets eukaryotic host cells, directly shaping infection outcomes. Specific effectors modulate innate immune signaling; reorganize the cytoskeleton; damage cellular membranes; and promote bacterial invasion, persistence, and dissemination within host tissues (Weber et al. 2016; Fitzsimons et al. 2018; Lewis et al. 2019). By impairing immune cell function and suppressing inflammatory responses, the T6SS enables *A. baumannii* to evade detection and clearance, fostering conditions conducive to chronic or recurrent infection.

The functionality of the T6SS relies on several conserved structural components. The hemolysin-coregulated protein (Hcp) polymerizes to form the inner tube, with its secretion serving as a hallmark of an active system and being indispensable for effector delivery (Carruthers et al. 2013). The valine-glycine-repeat G protein (*VgrG*) forms the spike tip, frequently carrying or interacting with effectors to penetrate target cell membranes (Weber et al. 2016). Additional accessory components, such as *TagX*, contribute toxins, assist in apparatus assembly, and increase firing efficiency (Fig. 3).

The ecological and clinical significance of the T6SS is considerable (Lin et al. 2021; Mijatović Scouten et al. 2025). Its expression is often tightly regulated: some strains repress T6SS activity during host infection to minimize immune detection and only upregulate T6SS activity during competitive encounters with other bacteria (Bhowmik et al. 2025; Lin et al. 2021). T6SS-positive strains consistently exhibit greater environmental fitness, hospital persistence, and virulence in animal models (Repizo et al. 2015; Lucidi et al. 2024; Lin et al. 2021). When the T6SS genes are disrupted, the bacterium loses much of its ability to cause infection and compete with other microbes (Dong et al. 2013; Repizo et al. 2015). This reduction in virulence and competitiveness highlights the T6SS as a promising target for developing new antibacterial treatments (Lin et al. 2021; Lucidi et al. 2024).

#### Type IV secretion system (T4SS)

Unlike the T6SS, the Type IV Secretion System (T4SS) is notable for its ability to both transfer genetic material, such as plasmids, and deliver effector proteins directly into other cells, whether bacterial or eukaryotic. This versatility not only promotes the spread of genes between organisms but also plays an important role in the disease-causing ability of *A. baumannii*. By facilitating the rapid acquisition of new genetic traits, including antimicrobial resistance, the T4SS plays a central role in an organism’s adaptability and persistence in challenging environments (Iruegas et al. 2023; Backert and Meyer 2006, Juhas et al. 2008).

One of the most critical functions of the T4SS is the conjugative transfer of plasmids carrying resistance and virulence genes. Through this mechanism, *A. baumannii* can share genetic material both with its own kind and with other bacterial species. This process has been directly linked to the spread of plasmids carrying extended-spectrum β-lactamase and car*bap* enemase genes, which has contributed to major hospital outbreaks (Liu et al. 2014). The result is accelerated evolutionary change, enabling the rapid emergence of multidrug-resistant lineages that are notoriously difficult to treat.

In addition to DNA transfer, the T4SS is capable of secreting effector proteins that directly manipulate host cell biology. These effectors can subvert immune signaling pathways, alter cytoskeletal dynamics, and influence apoptosis, thereby creating intracellular conditions that favor bacterial survival and replication. These functions not only increase virulence but also facilitate tissue colonization and chronic infection (Fig. 3).

The operation of the T4SS depends on a set of conserved genetic components. The *tra* (transfer) genes produce structural proteins that are vital for assembling and maintaining the conjugation machinery. At the core of this system are the *VirB1–VirB11* proteins, supported by the coupling protein *VirD4*. Together, they modulate the formation, control, and energy-driven transfer of DNA and effector molecules during the conjugation process (Redzej et al. 2017). Together, these elements create a versatile molecular conduit capable of handling a wide range of substrates.

In clinical settings, the T4SS greatly boosts *A. baumannii*’s ability to adapt and persist. By enabling the horizontal transfer of resistance genes and traits that improve survival, it speeds up the appearance of new, more dangerous strains that can spread rapidly in hospitals. Its effector-mediated modulation of host processes likely contributes to immune evasion and persistence, further cementing its role in pathogenesis.

When considered alongside the T6SS, the T4SS highlights the multifaceted survival strategy of *A. baumannii*. The T6SS ensures dominance through interbacterial antagonism and direct interference with host defenses, whereas the T4SS drives gene flow and fine-tunes host‒pathogen interactions. Together, these systems provide complementary advantages, enabling rapid adaptation, long-term persistence, and resistance to both antimicrobial interventions and host immunity. This synergistic interplay underlies *A. baumannii’s* reputation as one of the most formidable hospital-acquired threats worldwide.

The T4SS dramatically advances the adaptability of *A. baumannii*. The rapid acquisition and dissemination of resistance and fitness genes is instrumental in the constant emergence of new, more dangerous strains in clinical settings. T4SS-mediated effectors may also contribute to tissue colonization, immune evasion, and chronic infection.

Both the T6SS and T4SS are critical for *A. baumannii*’s dominance in healthcare environments and its success as opportunistic pathogens. The T6SS facilitates interbacterial competition and supports evasion of host defenses, whereas the T4SS is pivotal for gene flow and pathogenesis. The synergistic effects enable *A. baumannii* to adapt rapidly, persist, and evade both conventional treatment and host immunity, contributing directly to its notoriety as a high-priority hospital-acquired threat.

### Iron acquisition systems

**Fig. 4:**
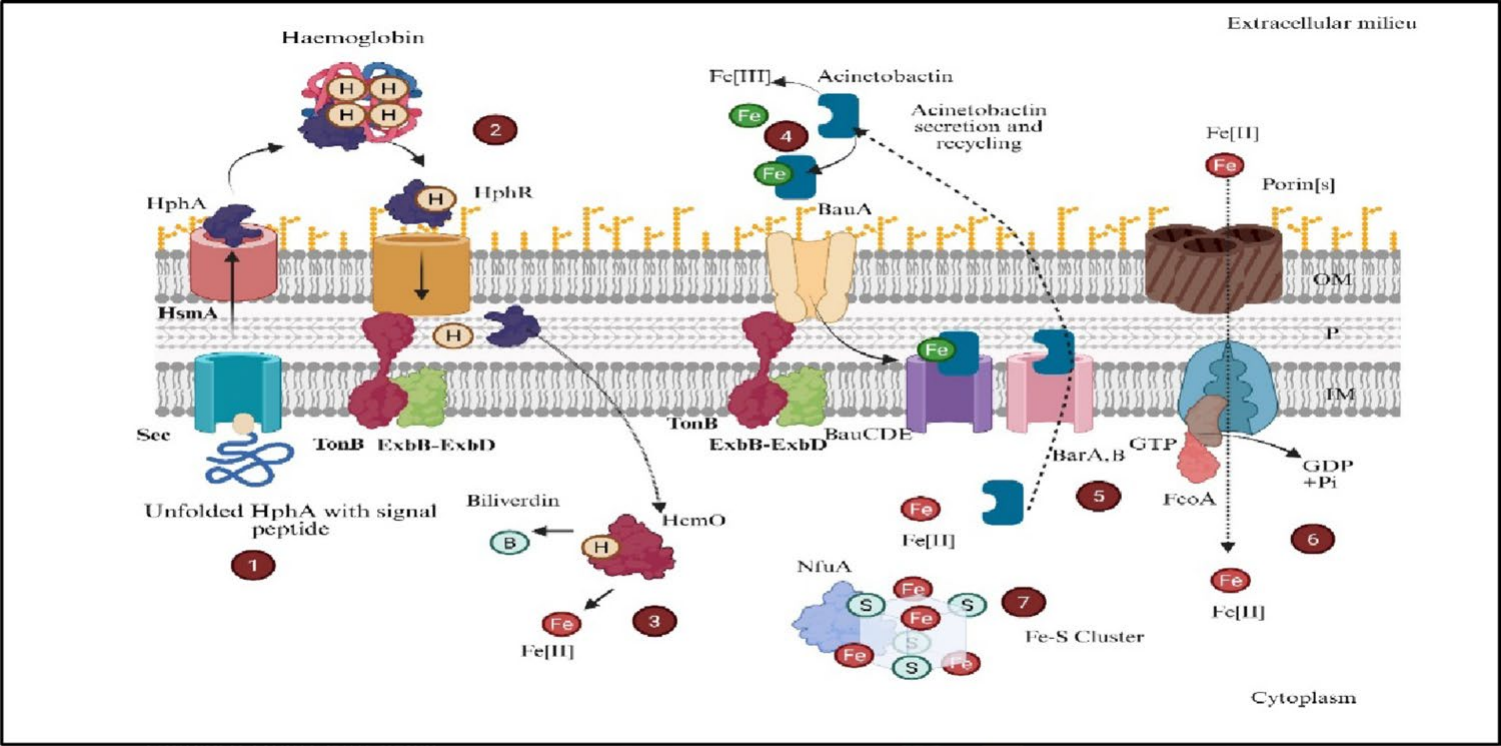
Overview of iron acquisition and heme utilization mechanisms in Acinetobacter species. The diagram illustrates multiple coordinated pathways enabling efficient uptake, transport, and intracellular utilization of iron under limiting conditions. (1) Heme-binding proteins such as HphA are translocated across the inner membrane via the Sec secretion system. (2) Hemoglobin-derived heme is captured at the cell surface by HphR and transported through TonB–ExbB–ExbD–dependent energy transduction. (3) Once internalized, the heme oxygenase HemO degrades heme into biliverdin and releases Fe(II). (4) Siderophore-mediated uptake is mediated through the acinetobactin pathway, where Fe(III)-bound acinetobactin complexes are recognized by the outer membrane receptor BauA and transported via BauCDE with TonB energy support, followed by siderophore recycling. (5) Free Fe(II) may additionally enter via porin-associated transport and the Feo system (FeoA/FeoB) driven by GTP hydrolysis. (6) Cytoplasmic Fe(II) pools are made available for metabolic processes. (7) Iron is incorporated into intracellular Fe–S clusters via scaffold proteins such as NfuA, supporting essential cellular functions, including respiration, DNA repair, and oxidative stress response. Collectively, these systems enable Acinetobacter spp. to successfully compete in iron-restricted host environments, contributing to survival, virulence, and persistence. Created in BioRender. Aranjani (2025) https://BioRender.com/9fwuslj

Iron is vital for virtually all bacteria and functions as a cofactor in processes such as respiration, DNA synthesis, and defense against oxidative stress. Within the mammalian host, free iron is scarce and sequestered by proteins such as transferrin, lactoferrin, ferritin, and heme, creating a bottleneck that restricts microbial growth (Fig. 4). To overcome this nutritional immunity, *A. baumannii* has evolved multiple overlapping iron-scavenging strategies that ensure iron uptake under diverse environmental conditions. Broadly, these systems include (1) high‐affinity siderophore production and uptake; (2) dedicated ferrous iron transporters; and (3) heme/hemin acquisition pathways (Fiester et al. 2016b).

#### Siderophores

Siderophores (metallophores) are small, high-affinity iron-chelating metabolites secreted by bacteria to acquire iron, a critical nutrient for survival and virulence during infection. Beyond their biological role, siderophores have gained attention as promising targets for novel antibacterial therapies. Strategies aimed at disrupting siderophore biosynthesis, transport, or function can effectively starve bacteria of iron, thereby attenuating their growth and virulence. Additionally, siderophores serve as natural delivery vehicles for a cutting-edge class of “Trojan horse” antibiotics, wherein antimicrobial agents are conjugated to siderophores to hijack bacterial iron uptake systems for targeted drug delivery. This approach enhances antibiotic efficacy against multidrug-resistant pathogens while minimizing off-target effects on the host microbiome (Kim et al. 2025; Gräff and Barry, 2024).

##### Acinetobacter β-actin

Acinetobactin is the primary siderophore that mediates iron acquisition during infection. The acinetobactin locus encodes enzymes for the biosynthesis (*BasA–BasJ*), export (*BarA/BarB*), and uptake (*BauA–E*) of two pH-dependent isomers: pre‐acinetobactin (favored at acidic pH) and acinetobactin (neutral/basic pH) (Penwell, 2013, Kim et al. 2021). Under iron‐starved conditions, both in vitro and during murine bacteremia, the entire acinetobactin cluster is upregulated, reflecting its essential role in overcoming host iron withholding. Genetic inactivation of acinetobactin biosynthesis (e.g., *ΔbasG*) drastically impairs growth in human serum, transferrin, and lactoferrin as sole iron sources and leads to a > 1,000‐fold reduction in bacterial burdens across organs in mouse infection models (Sheldon & Skaar 2020). The high conservation of the acinetobactin locus among clinical isolates underscores its indispensability for virulence.

##### Additional siderophores and functional redundancy

*A. baumannii* can synthesize up to ten siderophores, acinetobactin, baumannoferrins A/B, and fimsbactins A–F—from three distinct loci. While disrupting all biosynthetic pathways is required to abolish siderophore activity in iron-depleted media, only the loss of acinetobactin alone suffices to cause marked attenuation of serum‐based growth and in vivo virulence. Baumannoferrin and fimsbactins play a role in binding iron under laboratory conditions; however, their absence does not greatly affect bacterial survival in mouse models of bloodstream infection (Yang and Wencewicz, 2022, Mihara et al. 2004; Cook-Libin et al. 2022). This suggests that acinetobactin serves as the main siderophore that supports *A. baumannii* survival within the host environment.

#### Ferrous iron transport (Feo system)

The Feo system mediates the uptake of ferrous iron (Fe²⁺) and comprises FeoA (cytosolic accessory protein), FeoB (inner-membrane GTPase/transporter), and sometimes FeoC (regulator). Although deletion of *feoB* does not impair *A. baumannii* growth in iron‐limited media under aerobic conditions, where Fe²⁺ is scarce, it does reduce survival in human serum and increases susceptibility to complement‐mediated killing (Cartron et al. 2006; Lau et al. 2016; Runci et al. 2019). This phenotype suggests a role for FeoB in countering host antimicrobial peptides or oxidative stress during bloodstream infection, thereby contributing to overall pathogenicity even if it is dispensable for iron acquisition in vitro (Fig. 4).

#### Heme/hemin uptake

The *BasD*-dependent pathway allows the uptake of heme and hemin, which are abundant iron sources bound within host hemoproteins. *BasD* forms part of a complex transport system that extracts heme from hemoglobin and passes it through periplasmic binding proteins into the bacterial cytoplasm. Strains lacking the ability to take up heme show reduced virulence in animal models, indicating that heme serves as an important alternative source of iron during systemic infection (Cook-Libin et al. 2022) (Fig. 4).

### Enzymatic virulence factors

*A. baumannii* produces numerous hydrolytic enzymes that help in the breakdown of host macromolecules, allow nutrient uptake, aid tissue colonisation and immune evasion (Monem et al. 2020; Harding et al. 2018). The main enzymes that compose this mechanism include lipases, especially with concern for phospholipases, proteases, and multifuctional ß-lactamases. The whole set of enzymes boosts the bacterium’s defense and survival mechanisms, so it can persist and efficiently spread across the host organism (Wan et al. 2024).

#### Lipases and phospholipases

Lipases in *A. baumannii* hydrolyze ester bonds in host lipids, primarily targeting membrane phospholipids to release fatty acids and glycerol, which can serve as carbon and energy sources. This enzymatic function not only supplies nutrients but also breaches the integrity of membranes, allowing for the intake of bacteria into host cells and improving tissue penetration. One such extensively characterized example is *LipA*, a type II secretion–dependent lipase that is critical for growth on long-chain fatty acids and for maximal virulence in vivo. Mutants lacking *LipA* show severely impaired survival in bloodstream infection models and are significantly outcompeted by wild-type strains in spleen and liver colonization assays (Johnson et al. 2016). Similarly, the phospholipase C isoforms Plc1 and Plc2, encoded by *plc1* and *plc2*, cleave phosphatidylcholine in eukaryotic membranes, leading to hemolysis and cytotoxicity. Both genes are expressed under the condition of iron starvation, and there is no hemolytic activity for the double-knockout mutants. Among these, Plc1 appears particularly essential for virulence by evidence from insect and also mammalian infection models (Fiester et al. 2016; Kareem et al. 2017).

#### Proteases

Proteases secreted by *A. baumannii* play critical roles in host tissue colonization by degrading structural proteins and immune effectors, thereby releasing amino acids and peptides while dismantling host defenses. The zinc-dependent metalloprotease *CpaA*, which is secreted via the type II secretion system, selectively cleaves O-linked glycoproteins involved in complement activation and coagulation. In the absence of *CpaA*, reduced killing occurs in insect infection assays and reduced bacterial spreading occurs in mouse pneumonia, indicating its critical role in evading the immune system and distributing across the body (Florencia Haurat et al. 2020). In addition to metalloproteases, *A. baumannii* also produces different serine proteases and other peptidases. Comparative proteomic analyses of secretion-deficient mutants have revealed that these enzymes likely contribute to extracellular matrix degradation, cytokine inactivation, and disruption of epithelial barriers (Harding et al. 2018; Haurat et al. 2020).

#### β-Lactamases with dual roles

While β-lactamases are traditionally recognized for their role in hydrolyzing β-lactam antibiotics, certain enzymes in *A. baumannii* exhibit dual functions that extend beyond antimicrobial resistance to immune evasion. Notably, class D OXA-type carbapenemases such as *OXA-23* and *OXA-24/40* not only degrade a broad spectrum of β-lactams but also have been shown to inactivate cationic antimicrobial peptides (AMPs) in vitro, thereby enhancing bacterial survival via innate immune mechanisms (Silva & Domingues 2016; Lin et al. 2014; Piedra-Carrasco et al. 2017). Other class C and D β-lactamases also bind and cleave AMPs, which reduce their membrane disruption activity. Loss of enzymes makes bacteria more susceptible to host peptides, directly relating antibiotic determinants of resistance and virulence (Ferrari et al. 2008, Pérez-Llarena and Bou 2009, Brown and Livesay 2015).

Compared together, these enzymatic tactics demonstrate *A. baumannii’s* ability to modify metabolism but also have an impact on the host. Lipases and phospholipases penetrate host barriers for the acquisition of nutrients, proteases abolish immune protection and structural barriers, and β-lactamases provide defense against both the antibiotic and the natural immune systems. Understanding these mechanisms opens new avenues for antivirulence strategies targeting enzyme active sites, secretion pathways, or their regulatory networks (Monem et al. 2020; Harding et al. 2018).

### Stress tolerance mechanisms

*A. baumannii* encounters a variety of hostile stressors within the host environment, including reactive oxygen species (ROS) produced during phagocytosis, elevated febrile temperatures, and antimicrobial peptides. To survive these challenges, the bacterium employs an integrated network of defense mechanisms comprising antioxidant enzymes, molecular chaperones, and regulatory systems that detect and respond to stress, thereby ensuring its persistence and pathogenicity. Collectively, these adaptive responses enable *A. baumannii* to maintain viability under hostile host conditions, reinforcing its capacity to persist in clinical settings and contribute to chronic and recurrent infections.

#### Antioxidant enzymes

Antioxidant enzymes comprise the first defense mechanism against oxidant damage from immune cells. When immune cells such as neutrophils and macrophages phagocytize bacteria, they also activate NADPH oxidase complexes that yield damaging superoxide radicals (O₂⁻) (Staerck et al. 2017). *A. baumannii* defends itself against such radicals with superoxide dismutases (SODs) and catalases in a series of reactions (Manni et al. 2011). The sodA gene produces a Cu/Zn-dependent SOD that rapidly converts superoxide radicals to hydrogen peroxide (H₂O₂) and oxygen and also defends against damage to DNA, proteins, and fats. Other SODs, such as cytoplasmic *SodB* (Fe/Mn SOD) and periplasmic *SodC* (Cu/Zn SOD), also help remove these toxic entities from other loci within the cell. Notably, if the *sodA or sodB* genes are knocked out, the bacteria are superoxide-sensitive, move poorly, and have reduced virulence in infections in insects and in mammals (Heindorf et al. 2014; Steimbrüch et al. 2022).

Once superoxide dismutation occurs, the resulting hydrogen peroxide is destroyed by peroxidases and catalases to form water and oxygen. The catalase-peroxidase formed by *katG* plays a significant role in this degradation, whereas *katE* produces a single-function catalase that is important for the survival of stationary-phase bacteria for protracted intervals of oxidative stress (Sato et al. 2019; Sun et al. 2016). Deletion of both the *katG* and the *katE* abolishes hydrogen peroxide resistance, disables survival in serum, and significantly reduces virulence in experimental animals, illustrating just how critical these enzymes are for protection against oxidative stress (Sato et al. 2019).

#### Heat shock proteins

Heat shock proteins (HSPs) help keep proteins safe when temperatures rise, during exposure to harmful peptides, and under stress that can damage proteins. The *DnaK–DnaJ–GrpE* complex (Hsp70 system) connects to new or wrongly shaped proteins, stopping them from clumping together and helping them fold back into shape using energy from ATP. When this system is more active, bacteria can survive better in high heat and stressful conditions (Susin et al. 2006; Otvos et al. 2000; Dalbanjan et al. 2024). Likewise, the *GroEL–GroES* chaperonin complex (Hsp60/Hsp10) surrounds unfolded proteins in a double-ring space to help them refold with the help of ATP, keeping important enzymes working even under stress (Otvos et al. 2000; Wang et al. 2021; Tang et al. 2008). Other helper proteins, like the small heat shock proteins *IbpA/B* and the *ClpB* disaggregase, work together with *Hsp70* and *GroEL* to break apart protein clumps. These helpers are very important when water is low, as they protect proteins until better conditions come back.

#### Regulatory networks

The coordination of these stress responses is controlled by environmental cue-sensitive regulatory networks. The transcriptional controller OxyR senses hydrogen peroxide stress and activates the expression of *katG*,* katE*, and other antioxidants to rapidly suppress oxidative bursts. The alternative sigma factor *RpoH* (σ³²) recruits the transcription of chaperone genes like *dnaK* and *groEL* in response to heat shock and proteotoxic stress, achieving timely protection protein synthesis. The BfmRS two-component system, in turn, although mainly characterized for its role in biofilm control, also regulates stress tolerance genes and connects envelope integrity, desiccation resistance, and overall stress adaptation pathways. Such stress tolerance mechanisms enable *A. baumannii* to survive within phagocytes, remain alive on desiccated surfaces, and withstand robust conditions within the host (Palethorpe et al. 2022; Geisinger et al. 2018). This property renders treatment more complicated and enables the bacteria to thrive as a hospital-associated infection. Treatments that aim at the antioxidant defense mechanisms (such as SOD or catalase inhibitors), molecular chaperone systems (such as Hsp70 inhibitors), or their regulative pathways may render *A. baumannii* more amenable for removal from the immune system and increase efficiency for antimicrobial therapies (Cardoso et al. 2010; Palethorpe et al. 2022; Geisinger et al. 2018; Maharjan et al. 2023).

### Quorum sensing (QS)

Quorum sensing is a cell density–dependent communication mechanism that enables *A. baumannii* populations to synchronize gene expression and coordinate collective behaviors essential for survival, persistence, and virulence. Central to QS in *A. baumannii* is the *AbaI–AbaR* circuit: *AbaI* synthesizes N-acyl homoserine lactones (AHLs), primarily N‐hydroxydodecanoyl‐L‐homoserine lactone, which diffuse freely into the environment. As the bacterial population grows, the extracellular AHL concentration increases, and upon reaching a threshold, AHLs bind the LuxR‐type receptor AbaR. Ligand‐activated AbaR then directly or indirectly modulates the transcription of more than 300 genes, orchestrating behaviors from biofilm assembly to secretion system deployment and metabolic rewiring (Niu et al. 2008; Saipriya et al. 2020; Law and Tan 2022).

#### Quorum sensing mediated regulation of biofilm formation

QS upregulates key biofilm-related genes, including the *csu* chaperone–usher pilus operon and *pgaABCD* exopolysaccharide synthesis cluster, as well as the *bap* gene encoding a biofilm‐associated protein. Transcriptomic profiling of an *abaI* deletion mutant in ATCC 19,606 revealed the downregulation of 124 genes, including 9 type VI secretion system (T6SS) loci (*hcp*, *vgrG*, and *tss* clusters) and multiple biofilm matrix genes, which correlated with a marked reduction in biofilm biomass. This coordinated repression of adherence structures and matrix components underscores QS as a master regulator of surface‐associated communities that protect against desiccation, antibiotics, and host immunity (Bhargava et al. 2010; Cui et al. 2025).

#### Control of surface motility

QS also influences swarming and twitching motilities by modulating the expression of genes encoding type IV pili assembly (e.g., the *pilS/pilR* two-component regulatory system) and flagellar‐like structures, enabling population‐level exploration and colonization of new niches. The synchronized upregulation of motility genes at high cell densities promotes efficient surface migration, facilitating microcolony formation such that seeds mature biofilms (Xiong et al. 2022; Cui et al. 2025).

#### Expression of virulence determinants

In addition to structural factors, QS drives the expression of enzymatic and secretion-based virulence factors. Phospholipase C isoforms (*plc1*, *plc2*), which disrupt eukaryotic membranes to aid invasion, are induced by AbaR, as are genes for proteases, siderophore receptors (e.g., *bauA*), and class D β-lactamases, integrating offense and defense strategies under QS control. Loss of QS signaling attenuates cytotoxicity, T6SS‐mediated interbacterial competition, and invertebrate virulence, highlighting its broad impact on pathogenic potential.(Xiong et al. 2022; Pathoor et al. 2024; Cui et al. 2025).

#### Fine-tuning by AbaM and global regulators

QS output is buffered by the adjacent *abaM* gene, a negative regulator whose deletion yields hyper-QS phenotypes with elevated AHL levels, enhanced biofilm and motility gene expression, and paradoxically reduced virulence in animal models, suggesting that balanced QS activity is critical for optimal pathogenic fitness. Furthermore, QS interacts with global regulatory systems such as BfmRS, linking cell envelope stress responses, desiccation tolerance, and antibiotic resistance to population‐level signaling. (Farrow et al. 2018; Xiong et al. 2022)

#### Therapeutic targeting of quorum sensing

Given the centrality of QS to *A. baumannii* virulence, quorum-quenching approaches enzymatic AHL degradation, AHL‐analog inhibitors, or direct *AbaI/AbaR* antagonists, offer promising strategies to disrupt biofilm formation, dampen secretion systems, and sensitize bacteria to antimicrobial and host defenses without imposing strong selective pressure for resistance.(Evans et al. 2021; López-Martín et al. 2021).

### Motility

In contrast to earlier assumptions that *A. baumannii* is strictly non-motile, this pathogen employs sophisticated, surface‐associated movement strategies, namely, twitching motility driven by type IV pili (T4P) and flagellum-independent sliding or “surface‐associated” motility that are integral to colonization, biofilm development, and virulence.

#### Twitching motility via type IV pili

Type IV pili (T4P) are long, flexible, retractable filaments approximately 6–8 nm in diameter that are composed primarily of the major pilin *PilA*, with several minor pilin proteins contributing to pilus stability, function, and host interaction. Pilus assembly is mediated by a multiprotein complex spanning the inner and outer membranes, including the ATPase *PilB* (extension motor), the inner membrane platform protein *PilC*, the prepilin peptidase *PilD*, the retraction ATPases *PilT* and *PilU*, and the outer membrane secretin PilQ (Craig et al. 2004; Clemmer et al. 2011). PilB-driven polymerization of *PilA* extends the pilus outward, enabling surface adhesion through tip-associated adhesins, whereas *PilT/PilU*-driven depolymerization generates retraction forces of up to 100 pN, pulling the bacterial cell forward in discrete ~ 1 μm “twitch” steps.

Genetic analyses highlight the dual requirements for pilus extension and retraction in terms of motility and competence. Mutations in *pilA*,* pilD*,* or pilT* abolish twitching and natural transformation, whereas *pilT* mutants exhibit hyperpiliation but are incapable of retraction, resulting in static surface attachment without directional movement (Harding et al. 2013). Functionally, T4P-mediated twitching facilitates initial surface exploration, enabling bacteria to locate and colonize favorable niches. This early-stage motility promotes irreversible attachment and microcolony formation, which subsequently results in the formation of structured biofilms through interactions with Csu chaperone–usher pili and exopolysaccharide components such as biofilm-associated protein (*Bap*) and poly-β−1,6-N-acetylglucosamine (PNAG) (Carruthers et al. 2013; Ronish et al. 2018). Thus, T4Ps not only act as locomotory organelles but also serve as multifunctional devices linking motility, adherence, DNA uptake, and biofilm development.

#### Surface-associated motility

In addition to twitching, *A. baumannii* exhibits a pili-independent form of locomotion termed sliding, or surface-associated motility, which occurs on semisolid surfaces (0.5–1% agarose). Unlike T4P-driven movement, sliding relies on passive outward expansion powered by a surfactant-mediated reduction in surface tension. This motility mode is facilitated by secreted amphipathic molecules, including 1,3-diaminopropane, whose production is linked to modifications in lipooligosaccharide (LOS) composition and the activity of resistance–nodulation–division (RND) efflux pumps such as *AdeIJK*. These pumps not only export biosurfactants but also secrete quorum sensing (QS) signal molecules, linking sliding motility to population-level regulation (Blaschke et al. 2021; Corral et al. 2021; Skiebe et al. 2012).

Environmental conditions strongly affect sliding efficiency. Optimal motility is observed under iron-limited conditions, neutral pH, and ambient light exposure, suggesting the complex integration of metabolic and environmental signals. The two-component regulatory system BfmRS plays a pivotal role in sensing these conditions. As an envelope stress and light-responsive regulator, BfmRS modulates the transcription of motility-related genes, including those involved in surfactant biosynthesis and LOS remodeling (Craig et al. 2004; Harding et al. 2013; Kim et al. 2022a).

#### Regulatory networks of motility

Surface motility in *A. baumannii* emerges from the coordinated activity of two-component signal transduction systems, cyclic-di-GMP (c-di-GMP) second messenger signaling, and quorum sensing (Jeong et al. 2024; Blaschke et al. 2021).

The BfmRS two-component system exerts differential control over motility and biofilm formation. Deletion of the sensor kinase *bfmS* results in marked suppression of both twitching and sliding motility, which is attributable to downregulation of *pilT* and the *A1S_0112–A1S_0119* operon encoding the surfactant biosynthesis machinery. In contrast, deletion of the response regulator *bfmR* primarily compromises biofilm and pellicle formation through repression of the csu pilus operon while retaining motility. This functional divergence underscores how *A. baumannii* can differentially regulate adherence versus dispersal depending on environmental and physiological cues (Kim et al. 2022a).

C-di-GMP signaling acts as a central molecular switch between motile and sessile lifestyles. High levels of intracellular c-di-GMP, synthesized by diguanylate cyclases such as *A1S_1695*,* A1S_2506*, and *A1S_3296*, activate biofilm-associated loci, including csu pili and exopolysaccharide biosynthesis genes, while repressing T4P extension and surfactant secretion. Conversely, phosphodiesterases such as *A1S_1254* degrade c-di-GMP, derepressing motility programs and enabling both twitching and sliding activity (Xiong et al. 2022). This reversible modulation ensures that *A. baumannii* can transition rapidly between attachment and dissemination states in response to environmental fluctuations.

The *AbaI–AbaR* quorum sensing system integrates population density signals to synchronize motility behaviors across the bacterial community. At high cell densities, QS activation induces the expression of the *PilS/PilR* regulatory system and associated T4P assembly genes, promoting coordinated surface movement and the expansion of multicellular microcolonies (Ahmad et al. 2020). By coupling motility to cell–cell communication, *A. baumannii* ensures that surface colonization is a collective and structured process, maximizing both spatial coverage and biofilm robustness.

## Antibiotic resistance

**Fig. 5:**
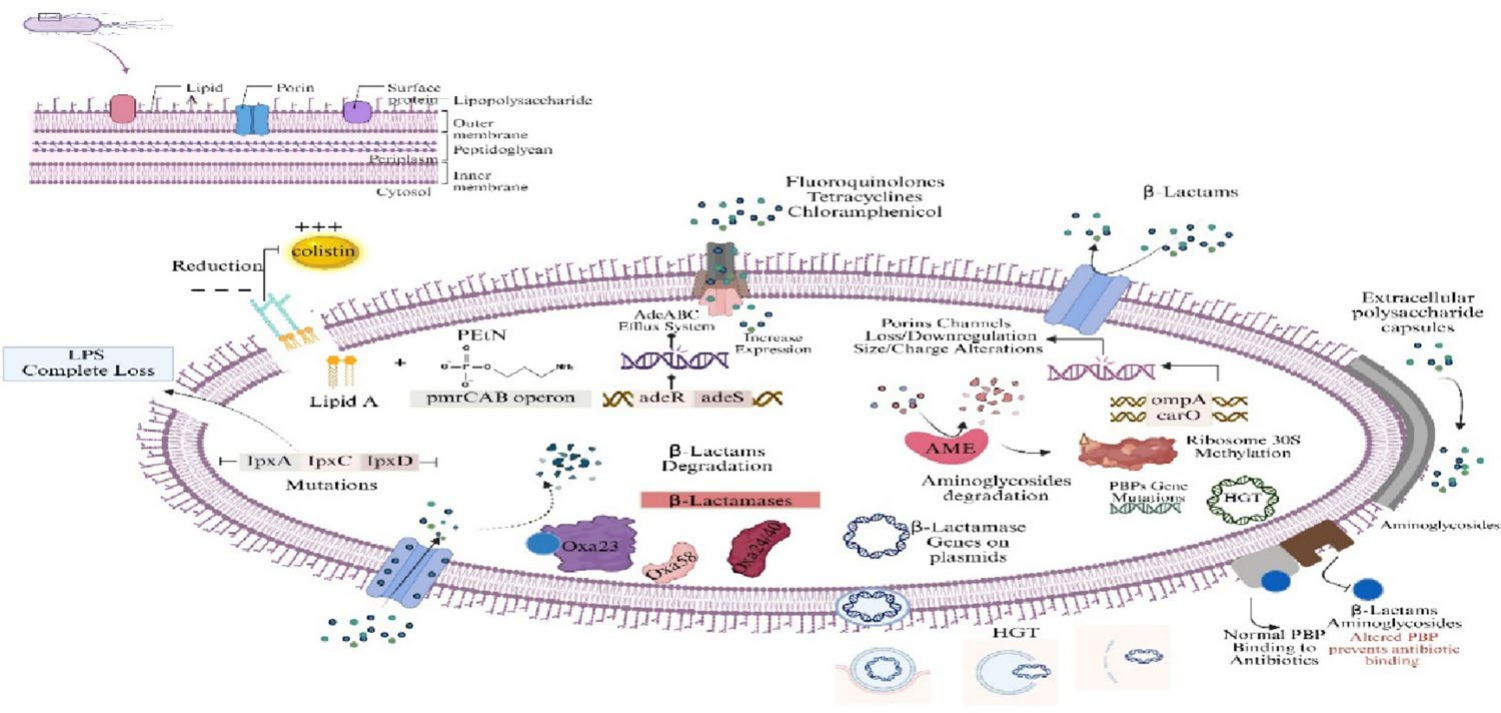
Mechanisms of antibiotic resistance in *Acinetobacter* *baumannii*. Resistance arises through β-lactamase production (including OXA-type enzymes), target site modifications such as altered PBPs and 30 S ribosomal methylation, aminoglycoside-modifying enzymes, efflux pump overexpression, porin loss or structural alterations, capsule-mediated protection, lipid A remodeling or LPS loss conferring colistin resistance, and horizontal gene transfer, collectively driving multidrug resistance. Created in BioRender. Aranjani (2025) https://BioRender.com/9fwuslj

Antibiotic resistance in *A. baumannii* not only ensures bacterial survival in the presence of antimicrobials but also amplifies its pathogenic potential, contributing significantly to persistence, adaptability, and the severity of infections. These resistance mechanisms operate as integrated survival strategies, simultaneously mitigating the effects of antibiotics and host immune assaults (Fig. 5).

### Beta-lactamases

*A. baumannii* produces a diverse repertoire of beta-lactamases, including class C (ampC) and class D (OXA-type) enzymes, as well as metallo-beta-lactamases such as NDM. These enzymes hydrolyze the beta-lactam ring of antibiotics such as penicillins, cephalosporins, and carbapenems, thereby nullifying their bactericidal activity. Genes such as *blaOXA-23*,* blaOXA-24*,* blaOXA-58*, and *blaNDM-1* are frequently detected in clinical isolates and are often mobilized by plasmids and insertion sequences, promoting rapid horizontal dissemination across strains (Wang et al. 2021).

Beyond simple drug inactivation, beta-lactamase production enables the bacterium to persist under intense therapeutic pressure within clinical environments, facilitating colonization of medical devices and chronic infection cycles. The co-expression of multiple beta-lactamases with other resistance determinants further strengthens persistence by producing multidrug-resistant (MDR) and extensively drug-resistant (XDR) phenotypes that are difficult to eradicate (Das et al. 2022; Vila et al. 2007; Peleg et al. 2008; Coyne et al. 2011).

### Efflux pumps as drivers of multidrug resistance

A major contributor to multidrug resistance and persistence in *A. baumannii* is the overexpression of resistance–nodulation–cell division (RND)-type efflux pumps, including *AdeABC*,* AdeIJK*, and *AdeFGH*. These tripartite complexes traverse both membranes and expel a wide range of compounds, including aminoglycosides, tetracyclines, fluoroquinolones, biocides, and host-derived antimicrobial peptides. This dual functionality reinforces both antibiotic resistance and immune evasion, allowing *A. baumannii* to survive within macrophages and epithelial barriers (Fig. 5).

Mutations in the regulatory genes (*adeRS*,* adeN*,* adeL*) result in constitutive efflux pump overexpression, enhancing the bacterium’s ability to adapt to harsh hospital conditions while simultaneously augmenting virulence through increased epithelial adherence and biofilm formation. Consequently, efflux pumps function as molecular shields that link drug resistance with host persistence (Nordmann et al. 2011; Xiong et al. 2022; Mack et al. 2025).

### Target site modifications

Alterations in antibiotic target sites further consolidate the survival of *A. baumannii* under antimicrobial and immune pressures. Mutations in penicillin-binding proteins (PBPs) decrease the affinity of beta-lactam antibiotics, while point mutations in *gyrA* and *parC* confer resistance to fluoroquinolones by altering DNA gyrase and topoisomerase IV binding sites. These target site modifications, while primarily conferring resistance, also enhance survival under host stress by minimizing interactions with immune effectors that exploit similar molecular pathways (Abdi et al. 2020; Wimalasekara et al. 2025).

### Carbapenem resistance

Carbapenem-resistant *A. baumannii* (CRAB) strains represent a critical clinical threat due to their extensive drug resistance and high persistence rates (Thacharodi et al. 2024; Kyriakidis et al. 2021). Resistance arises mainly from the acquisition of carbapenem-hydrolyzing OXA-type beta-lactamases such as *OXA-23*,* OXA-24*, and *OXA-58*, and to a lesser extent, metallo-beta-lactamases such as *NDM*,* VIM*, and *IMP* (Shi et al. 2024; Poirel and Nordmann 2006; de Souza et al. 2025). Additionally, the loss or downregulation of outer membrane porins such as *CarO* reduces drug permeability, while concurrent efflux pump activation synergistically limits intracellular antibiotic accumulation (de Souza et al. 2025; Kyriakidis et al. 2021).

Such multifaceted resistance not only confers protection from antibiotics but also enhances bacterial fitness in the host environment by reinforcing envelope stability and reducing susceptibility to complement-mediated killing. Consequently, these highly resistant clones frequently persist as endemic lineages within intensive care units (Poirel and Nordmann 2006; Singh et al. 2013; Wimalasekara et al. 2025) (Fig. 5).

### Genetic plasticity and persistence

The remarkable genetic plasticity of *A. baumannii* underpins its capacity to acquire and disseminate resistance and virulence determinants. Horizontal gene transfer (HGT) mediated by plasmids, transposons, and integrons enables the bacterium to continuously remodel its genome in response to selective pressures within healthcare environments. This dynamic adaptability not only facilitates resistance acquisition but also promotes long-term persistence by allowing *A. baumannii* to occupy diverse ecological niches, evade immune clearance, and endure prolonged antimicrobial exposure.

Together, these interlinked resistance mechanisms equip *A. baumannii* with extraordinary survival potential, establishing it as one of the most tenacious and clinically significant hospital-acquired pathogens of modern medicine.

## Genetic plasticity and horizontal gene transfer (HGT)

*A. baumannii* shows genetic plasticity, which enables it to survive in different environments, especially healthcare settings. This is achieved by mechanisms such as horizontal gene transfer (HGT) and mobile genetic elements (MGEs).

### Genetic plasticity, horizontal gene transfer, and mobile genetic elements

The remarkable genetic plasticity of *A. baumannii* underpins its adaptability, multidrug resistance, and persistence in healthcare environments. This flexibility is primarily driven by horizontal gene transfer (HGT) and the mobilization of mobile genetic elements (MGEs), which together enable the acquisition, rearrangement, and dissemination of genes associated with antimicrobial resistance and virulence.

Through HGT, *A. baumannii* incorporates exogenous genetic material into its genome via several mechanisms. Natural transformation allows the uptake and integration of free extracellular DNA, facilitating the acquisition of clinically significant resistance genes such as *blaNDM-1 and blaOXA-23* (Cavallo et al. 2023; Harding et al. 2018; Touchon et al. 2014). Conjugation, mediated by plasmids and conjugative elements, represents a dominant route for gene exchange, enabling the rapid spread of multidrug resistance determinants within and between bacterial species through specialized pili (Jeon et al. 2023; Mindlin et al. 2021; Wachino et al. 2019; Thomas & Nielsen 2005). Although less frequently reported, transduction mediated by bacteriophages can transfer chromosomal or plasmid-borne genes, contributing to the genomic diversification of clinical isolates (Harding et al. 2018). Additionally, outer membrane vesicle–mediated transfer provides a contact-independent means of exchanging DNA, further expanding the bacterium’s adaptive potential (Rumbo et al. 2011).

Mobile genetic elements (MGEs) act as dynamic vehicles for this genetic exchange. These include plasmids, transposons, integrons, resistance islands, and insertion sequences, each facilitating the capture and mobilization of antibiotic resistance genes (ARGs) across bacterial populations (Pagano et al. 2016; Cerezales et al. 2020; Noel et al. 2022; Correa et al. 2024; Salem et al. 2025). MGEs such as Tn7-like transposons and AbaR-type resistance islands play central roles in the dissemination of carbapenemase genes, notably *blaOXA-23*, driving the emergence of carbapenem-resistant *A. baumannii*. Complex resistance islands, including *AbaR*, integrate multiple MGEs and ARGs, often forming unique structural variants that enhance the bacterium’s multidrug-resistant (MDR) phenotype (Adams et al. 2010; Bi et al. 2020).

This extensive genetic interplay between HGT and MGEs provides *A. baumannii* with extraordinary genomic adaptability, enabling it to survive under intense selective pressures such as antibiotic exposure. Environmental and clinical stressors further accelerate this gene flow by promoting the integration of foreign DNA, particularly when defense systems like CRISPR-Cas are compromised (Partridge et al. 2018; Vrancianu et al. 2021; Yaikhan et al. 2024).

Collectively, these processes underpin the pathogen’s ability to rapidly evolve and persist in nosocomial environments. The growing prevalence of diverse MGEs among clinical isolates highlights the urgent need for genomic surveillance and containment strategies to curb the dissemination of resistance determinants and maintain the efficacy of antimicrobial therapies (see Table 1).

**Table 1:** Mobile genetic elements in *A*. *baumannii*

MGE type	Role in resistance	Examples	References
Plasmids	Carry and transfer ARGs across bacteria	Bla _*OXA−23*_ plasmids	(Wang et al. 2021; Salgado-Camargo et al. 2020; Ababneh et al. 2023)
Transposons	Mobilize and spread resistance genes	Tn7-like transposons	(Correa et al. 2024; Noel et al. 2022)
Integrons	Capture and express ARG-containing gene cassettes	Diverse integrons	(Deylam et al. 2017; Abed et al. 2024)
Resistance Islands	Harbor multiple, often diverse, resistance genes in large genomic segments	AbaR islands	(Hamidian et al. 2018; Šeputienė et al. 2011)
Insertion seÃquences	Enhance gene mobility and promote strong expression of resistance determinants	ISAba elements	(Vijayakumar et al. 2022; Lee et al. 2011)

## Conclusion and future perspectives

The virulence determinants of *A. baumannii* collectively underpin its capacity for clinical persistence, tissue colonization, immune evasion, and multidrug resistance. Key pathogenic attributes, including biofilm formation, tolerance to oxidative and thermal stress, efficient iron acquisition mechanisms, and the deployment of specialized secretion systems, confer exceptional adaptability within hospital environments. Moreover, its dynamic genome, enriched with mobile genetic elements such as insertion sequences, transposons, and plasmids, facilitates rapid horizontal gene transfer of resistance determinants including *blaNDM-1* and *blaOXA-23*, thereby complicating therapeutic interventions and accelerating resistance evolution (Vijayakumar et al. 2022; Sahl et al. 2011). This genomic plasticity not only underlies antimicrobial resistance but also enables the acquisition of virulence factors that modulate host interactions and promote environmental persistence.

Similar patterns of genomic flexibility and adaptability are observed in other clinically significant pathogens. Genome-sequencing studies of *Staphylococcus aureus* have revealed extensive horizontal gene transfer and mobile element diversity contributing to the evolution of methicillin resistance and virulence (Wisal et al. 2024; Holden et al. 2004). Likewise, *Corynebacterium pseudotuberculosis* Cp162 and other *Corynebacterium* species exhibit dynamic genomes with prophages, pathogenicity islands, and resistance genes that facilitate host adaptation and immune evasion (Hassan et al. 2012; Ali et al. 2013). These comparative studies corroborate the critical role that genome plasticity plays in shaping pathogen evolution, enabling rapid responses to selective pressures such as antibiotic exposure and host immune defenses.

Future investigations should move beyond descriptive characterization toward mechanistic and integrative analyses to delineate novel antivirulence targets. Approaches combining transcriptomics, proteomics, metabolomics, and host–pathogen interaction modeling hold promise for elucidating the complex regulatory networks governing virulence expression under clinically relevant conditions. Furthermore, structure-based drug discovery and integrative proteomics strategies, successfully utilized in multidrug-resistant bacterial species, can accelerate the identification and validation of small-molecule inhibitors targeting essential virulence regulators or structural components of secretion systems (Radusky et al. 2015; Wisal et al. 2024).

Translational directions focusing on antivirulence include the rational design of quorum-sensing blockers, biofilm disruptors, iron chelators, and efflux pump inhibitors. These interventions, guided by computational modeling and validated through omics-driven approaches, may synergize with next-generation antimicrobials and precision infection control to reduce the clinical burden of *A. baumannii*. Ultimately, a systems-level strategy that integrates multi-omics data, structure-based therapeutic development, and host-directed therapies will be vital to overcoming the remarkable resilience and evolutionary adaptability of this pathogen.

## Data Availability

No datasets were generated or analysed during the current study.
